# Orbital cellulitis following acute sinusitis in a neonate

**DOI:** 10.1590/0037-8682-0257-2025

**Published:** 2025-09-29

**Authors:** Elif Gozgec, Muhammed Mukremin Bugdaci

**Affiliations:** 1 Ataturk University School of Medicine, Department of Radiology, Erzurum, Turkey.

**FIGURE 1:**
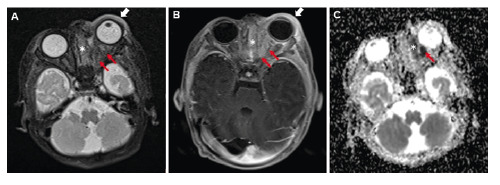
Axial section T2-weighted brain magnetic resonance imaging (MRI) **(A)** demonstrates diffuse intensity increases indicative of sinusitis in the ethmoid cells and medial rectus muscle. Furthermore, an increase in intensity and thickness is evident in the left periorbital area. Axial section T1-weighted brain MRI with contrast **(B)** reveals increased contrast enhancement in the left extraocular muscles, particularly noticeable in the rectus medialis and the intraconal-extraconal fatty planes. The apparent diffusion coefficient image **(C)** shows a hypointense signal, which indicates restricted diffusion in the ethmoid cells and the medial rectus muscle. (Ethmoid cells; asterisk, white arrow; periorbital area, red arrows; medial rectus muscle).

A patient, on the 10th postnatal day, was admitted to our clinic with fever, rhinorrhea, and swelling of the left eye. He had been born healthy at term via a cesarean section. Physical examination revealed periorbital edema, redness, elevated temperature in the left eye, and purulent nasal discharge. The white cell count was 26.63 × 10^3^/µL, and the C-reactive protein concentration was 10.6 mg/dL. *Staphylococcus aureus* was cultured from a nasal cavity wound. A lumbar puncture yielded negative results. Magnetic resonance imaging was performed to assess the preseptal/postseptal separation and potential abscesses related to orbital cellulitis. T2-weighted images showed increased thickness and intensity in the left periorbital area, ethmoidal cells, and left medial rectus muscle. Heterogeneity and enhanced contrast were noted in the left extraocular muscle and intraconal-extraconal fatty planes. These areas demonstrated diffusion restriction on diffusion-weighted images (Figure 1). Intravenous cefotaxime and vancomycin were administered following the diagnosis of orbital and periorbital cellulitis. By the 10th day of treatment, the neonate responded well and was discharged with specific recommendations.

Orbital cellulitis is an extremely rare and potentially fatal condition that affects newborns^1^. The most common pathogens involved are *Staphylococcus aureus* and Streptococcus spp.. Preseptal infections are treated with antibiotics, and surgery may be required in postseptal cases with a poorer prognosis. However, distinguishing these two conditions using only clinical and laboratory data is challenging. Radiological imaging plays a crucial role in differentiating periorbital and orbital cellulitis, identifying abscesses, and distinguishing these conditions from other postseptal processes^2^.
